# PRDX1 exerts a photoprotection effect by inhibiting oxidative stress and regulating MAPK signaling on retinal pigment epithelium

**DOI:** 10.1186/s12886-024-03489-4

**Published:** 2024-06-06

**Authors:** Xiao-Ying Wen, Na Yang, Yang Gao, Wei-na Ma, Yan Fu, Ren-fei Geng, Yue-ling Zhang

**Affiliations:** Department of Ophthalmology, Baoding NO.1 Central Hospital, Baoding, Hebei China

**Keywords:** Retinal pigment epithelium, Photoprotection, Mitogen-activated protein kinase signaling pathway, Peroxiredoxin 1

## Abstract

**Background:**

The purpose of this study was to investigate the photoprotection effect of peroxiredoxin 1 (PRDX1) protein in ultraviolet B (UVB) irradiation-induced damage of retinal pigment epithelium (RPE) and its possible molecular mechanism.

**Methods:**

ARPE-19 cell viability and apoptosis were assessed by MTT assay and flow cytometry, respectively. Real-time quantitative reverse transcription polymerase chain reaction (qRT-PCR) was used to detect the PRDX1 expression. The corresponding kits were employed to measure the levels or activities of lactate dehydrogenase (LDH), 8-hydroxy-2-deoxyguanosine (8-OHdG), reactive oxygen species (ROS), malondialdehyde (MDA), glutathione peroxidase (GSH-Px), superoxide dismutase (SOD). Western blotting was applied to examine PRDX1 expression and mitogen-activated protein kinase (MAPK) signaling pathway-related proteins.

**Results:**

After exposure to 20 mJ/cm^2^ intensity of UVB irradiation for 24 h, ARPE-19 cells viability was decreased, the leakage degree of LDH and 8-OHdG were increased, and cell apoptosis was elevated. The expression of PRDX1 was significantly down-regulated in UVB-induced ARPE-19 cells. The low expression of PRDX1 was involved in high irradiation intensity. Overexpression of PRDX1 increased cell activity, decreased cell apoptosis, and LDH as well as 8-OHdG leakage in UVB-induced ARPE-19 cells. In addition to alleviating UVB-induced cell damage, PRDX1 overexpression also inhibited UVB-induced oxidative stress (down-regulation of ROS and MDA levels, up-regulation of GSH-Px and SOD activities) and the activation of MAPK signaling pathway in ARPE-19 cells.

**Conclusion:**

PRDX1 exerts a photoprotection effect on RPE by attenuating UVB-induced cell damage and inhibiting oxidative stress, which can be attributed to the inhibition of MAPK signaling pathway activation.

**Supplementary Information:**

The online version contains supplementary material available at 10.1186/s12886-024-03489-4.

## Background

Retinopathy has become one of the mos*t* common diseases in China and around the world due to the advancement of electronic technology and the acceleration of aging. Age-related macular degeneration (AMD), a retinopathy, can lead to blindness and severe vision loss [1]. In recent years, the prevalence of AMD has been on the rise and increases with age. As shown in some studies, approximately 288 million people are predicted to be affected by AMD by 2040 [2, 3]. Although the clinical progression of AMD has not been fully understood, the degeneration of retinal pigment epithelium (RPE) is an important causative factor in the pathogenesis of AMD [4]. However, the exact cause of RPE cell degeneration is still unknown. Oxidative stress plays an important role in RPE cell apoptosis and the development of AMD [5]. Therefore, the search for markers that can inhibit oxidative stress in RPE is crucial to alleviate AMD.

Ultraviolet B (UVB), an ultraviolet light that absorbs wavelengths in the range of 280–320 nm, has certain physiological effects on human skin. Specifically, prolonged exposure to UVB will cause intense photodamage to the skin and retina [6]. Melanin which is abundant in RPE can absorb light and reduce light scattering. It plays a photoprotection role in RPE by protecting intraocular tissues from oxidative damage. However, after long-term exposure to sunlight, UVB radiation can cause damage to the retina and then promote the development of AMD [7, 8]. Besides, the photoprotection decreases with age, significantly increasing the risk of developing AMD [9]. As demonstrated by Heck, et al., UVB induces direct DNA damage and oxidative stress in RPE by up-regulating reactive oxygen species (ROS) and modulating endogenous antioxidants [10]. Additionally, an in vivo study of the entire retina has demonstrated that UVB radiation induces gene mutation and dysregulation of protein levels. The above studies suggest that UVB damage is associated with AMD [11]. Thus, inhibiting UVB-induced oxidative stress is essential for the alleviation of UVB-induced AMD lesions.

Peroxiredoxin (PRDX) 1 is an enzyme belonging to the PRDX family. PRDX1 has been reported to regulate oxidant sensitivity in a variety of pathological and stressful states [12]. Besides, it can exert an inhibitory effect on cellular damage through anti-ROS activity [13]. In addition, due to its antioxidant role, PRDX1 is involved in several pathological processes, such as tumor and renal ischemia/reperfusion injury [14, 15]. Moreover, a proteomic investigation reveals a significant reduction in PRDX1 expression in RPE after exposure to UVA radiation [16]. Nonetheless, the ability of PRDX1 to prevent UV-induced RPE damage has not been experimentally verified, and its exact mechanism of action remains unclear. Therefore, we hypothesized that PRDX1 could play a protective role by inhibiting UVB-induced oxidative stress response in ARPE-19 cells. To demonstrate the above hypothesis, we first induced ARPE-19 cell damage by UVB irradiation. Then, we evaluated the protective effects of PRDX1 on cell activity and oxidative stress through a series of experiments including 3-[4,5-dimethylthiazol-2-yl]-2,5 diphenyl tetrazolium bromide (MTT) assay and biochemical assay. Overall, the purpose of this study was to provide a novel molecular target for the treatment of AMD.

## Materials and methods

### Cell culture

ARPE-19 cells (GDC0323), the RPE cells, were obtained from the China Center for Type Culture Collection. ARPE-19 cells were cultured in Dulbecco’s Modified Eagle Medium (Thermo Fisher Scientific, USA) containing 10% fetal bovine serum (Thermo Fisher Scientific, USA) and 1% penicillin-streptomycin (Solarbio, Beijing, China) in an incubator at 37 °C, 5% CO_2_, and 95% humidity. Notably, the medium was changed every two days. After cell confluence, trypsin (0.25%)-ethylenediaminetetraacetic acid (EDTA, 0.02%) (Solarbio, Beijing, China) was used for digestion and passage.

### UVB-induced cell damage model

Cells in the logarithmic growth phase were irradiated with different doses of UVB (0, 5, 10, 15, 20 mJ/cm^2^) for 24 h to construct a UVB-induced cell damage model according to previous studies [17, 18]. During UVB irradiation, the medium was removed, and the cells were covered with a phosphate buffer solution. The cells were irradiated at a fixed distance and variable dose under an 8-watt UV lamp (305 nm) (Cole-Parmer, Shanghai, China).

### Transfection

Cells were inoculated into 6-well plates at a density of 3 × 10^5^ cells/well. When cells reached about 70% confluence, the cells were transfected according to the instructions of the Lipofectamine 3000 reagent (Thermo Fisher Scientific, USA). The ARPE-19 cells were randomly divided into four groups: the Control group: ARPE-19 cells received no treatment; the UVB group: ARPE-19 cells were treated with UVB irradiation at an intensity of 20 mJ/cm^2^; the UVB + vector group: ARPE-19 cells were transfected with a negative vector and then subjected to irradiation with UVB at an intensity of 20 mJ/cm^2^ for 24 h; the UVB + PRDX1 group: ARPE-19 cells were transfected with pcDNA3.1-PRDX1 overexpression vector and then irradiated with UVB at an intensity of 20 mJ/cm^2^ for 24 h.

### MTT assay

Cells (5 × 10^3^ cells/well) at logarithmic growth stage were seeded into 96-well plates. After overnight culture, the cells were treated for 0 h and 24 h and then subjected to MTT assay. Subsequently, 20 µL of MTT solution (5 mg/ml) was added to each well for 4 h of incubation. Next, each well was supplemented with 150 µL dimethyl sulfoxide. Then, the supernatant was discarded, followed by mixing at room temperature for 5 min. The absorbance value at 490 nm was measured by a microplate reader.

### Kit detection

A 6-well plate was picked up for the inoculation of cells (2 × 10^5^ cells/well). The cells were subjected to treatments following cell adherence. Upon cell confluence, we determined the levels or activities of lactate dehydrogenase (LDH), 8-hydroxy-2-deoxyguanosine (8-OHdG), ROS, malondialdehyde (MDA), glutathione peroxidase (GSH-Px), and superoxide dismutase (SOD) in cell culture supernatant according to the procedure of the corresponding kits (Nanjing Jiancheng Bioengineering Institute, Nanjing, China). All tests were performed in triplicate.

### Real-time quantitative reverse transcription polymerase chain reaction

Total cellular RNA was extracted using the TRIzol kit (Life Technologies, USA) and stored at -80 °C. Next, the RNA was reverse transcribed to cDNA in accordance with the instructions of the Reverse Transcription Polymerase Chain Reaction (PCR) Kit (TaKaRa, Japan). Real-time quantitative reverse transcription PCR (qRT-PCR) was performed as described in SYBR® Premix Ex TaqTM lI Kit (TaKaRa, Japan) to detect the mRNA expression level of PRDX1. Glyceraldehyde-3-phosphate dehydrogenase (GAPDH) was used as an internal control for sample normalization. The primer sequences were shown in Table 1. Data analysis was conducted using a 2^−ΔΔCt^ method.

**Table 1:** qRT-PCR primer sequences

Genes	Primer sequences
PRDX1	F 5’- CGGGCCTсTAGACTTCT-3’
	R 5’- TATG TCTTCAGGAAATGCTA-3’
GAPDH	F 5’- GAAGGTGAAGGTC GGAGTC-3’
	R 5’- GAAGATGGTGATGGGATTTC-3’

PRDX1, peroxiredoxin 1; GAPDH, glyceraldehyde-3-phosphate dehydrogenase

### Cell apoptosis assay

The early and late apoptosis rates in ARPE19 with different treatments were determined using an Annexin V-Fluorescein Isothiocyanate/Propidium Iodide Apoptosis Detection kit according to the the instructions of manufacturers (Invitrogen, USA). Briefly, the cells were resuspended in the binding buffer to a concentration of 10^6^ cells/ml. Then, 5 µL Annexin V-fluorescein isothiocyanate and propidium iodide were added, followed by incubation at room temperature for 15 min in the dark. Ultimately, the cell fluorescence was detected on FACScan by flow cytometry within 1 h. FlowJo software was used to analyze the flow data.

### Western blotting

Cells were lysed on ice for 30 min using radioimmunoprecipitation assay buffer (Beyotime, Shanghai, China) supplemented with 1 mM phenylmethanesulfonyl fluoride (Beyotime, Shanghai, China). Next, the supernatant was collected after centrifugation at 10,000 g for 10 min at 4 °C. The total cellular protein concentration was determined using the bicinchoninic acid kit. Sodium dodecyl sulfate polyacrylamide gel electrophoresis gels were prepared by adding separation gel and concentrate gel into a glass gel mold. The prepared gels were used to separate 30 µg of denatured protein samples and protein markers, and the voltage was set to 80 V. Upon full separation, the protein markers were dropped to the interface of the separation gel, and the voltage was increased to 120 V. Following electrophoresis, the proteins were transferred from the gel to a polyvinylidene fluoride membrane (Millipore) at a constant current of 200 mA for 2 h of trans-blot transfer. Upon transfer, the membrane was peeled off and blocked with 5% skimmed milk for 1 h after termination of the electrophoresis. The rinsed membrane was incubated with corresponding primary antibodies overnight at 4 ℃ in a shaker. The primary antibodies used included anti-PRDX1, anti-extracellular signal-regulated kinase (ERK)1/2, anti-p-ERK1/2, anti-c-Jun N-terminal kinase (JNK), anti-p-JNK, anti-p-38, anti-p-p38, and anti-GAPDH (Abcam, England). The protein bands on the membrane were visualized using a chemiluminescent solution (Sigma-Aldrich, USA) and photographed by a gel imaging system. Image-Pro Plus software was applied to quantify the grayscale values of each protein band. GAPDH was considered as an internal control to analyze the relative expression of the target proteins.

### Statistical analysis

All experimental data results were expressed as mean ± standard deviation and visualized using GraphPad Prism 9.0 (GraphPad Software, La Jolla, CA, USA). Statistical analysis was performed using SPSS 21. 0 software. A t-test was used for comparison between the two groups, and a one-way analysis of variance was utilized to compare the differences among multiple groups. *P* < 0.05 was the criterion for a statistically significant difference.

## Results

### Down-regulation of PRDX1 expression in UVB-induced ARPE-19 cells

**Fig. 1:**
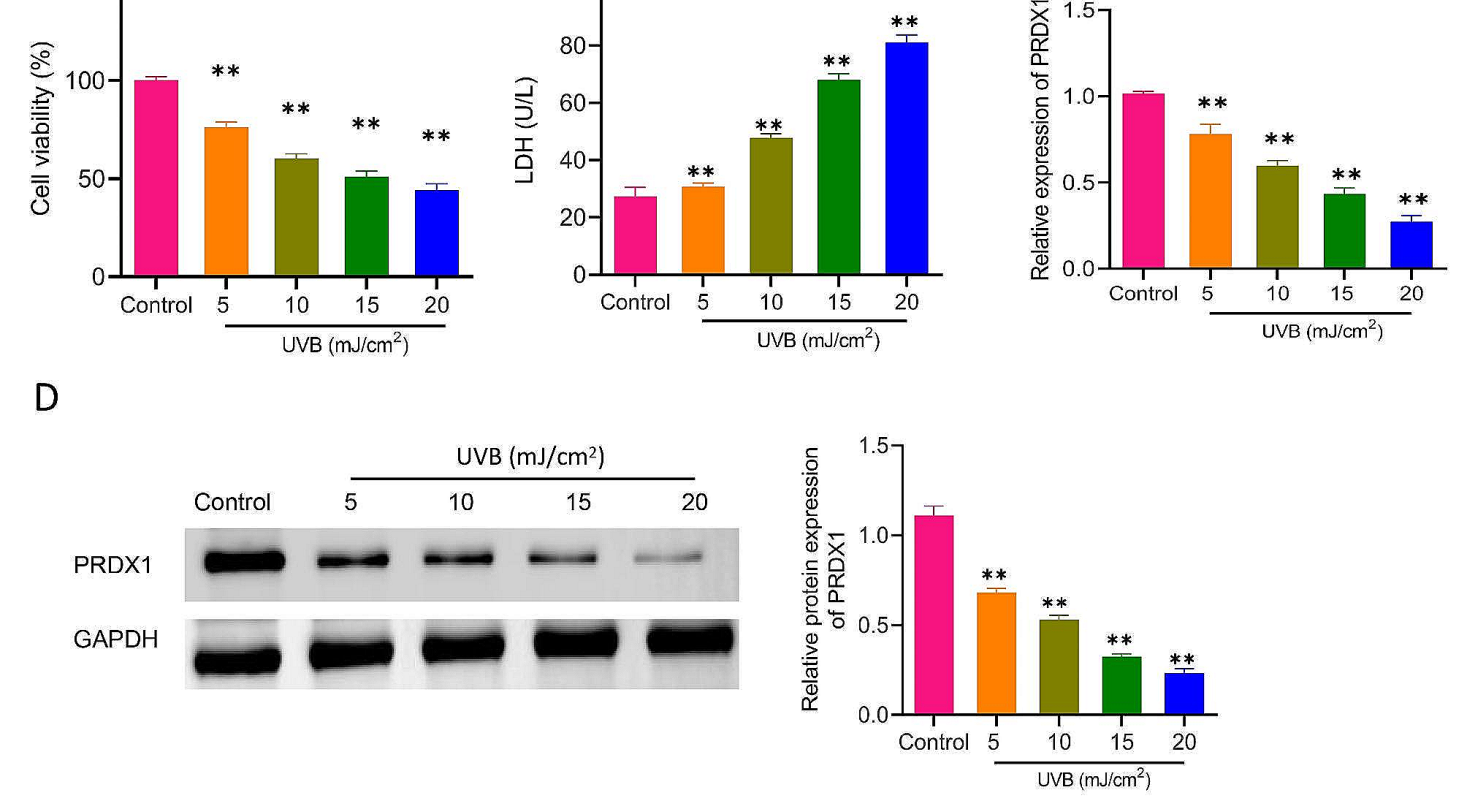
PRDX1 expression is down-regulated in UVB-induced ARPE-19 cells. **A**–**D**, MTT, biochemical kits, qRT-PCR and western blotting to analyze the effects of UVB irradiation with different intensities (5, 10, 15 and 20 mJ/cm^2^) on ARPE-19 cell viability (**A**), levels of LDH in cell culture supernatant (**B**), the relative mRNA levels of PRDX1 in cells (**C**) and protein expression levels of PRDX1 (**D**), **P < 0.01. PRDX1, peroxiredoxin 1; UVB: ultraviolet B; MTT, 3-[4,5-dimethylthiazol-2-yl]-2,5 diphenyl tetrazolium bromide; qRT-PCR, real-time quantitative reverse transcription polymerase chain reaction; LDH: lactate dehydrogenase

To explore the role of PRDX1 in UVB-induced cellular photodamage, ARPE-19 cell damage was induced by UVB irradiation. The ARPE-19 cells were treated with UVB at different intensities (5, 10, 15 and 20 mJ/cm^2^) to evaluate the effects of varied UVB intensities on the viability and toxicity of ARPE-19 in RPE. The results showed that ARPE-19 cell viability was significantly inhibited by UVB (*P* < 0.01, Fig. 1A). Compared with the Control group, cell viability was decreased by 24%, 40%, 49%, and 56% at 5, 10, 15 and 20 mJ/cm^2^, respectively. In addition, LDH levels were markedly increased by UVB irradiation in the culture supernatant of ARPE-19 cells, with 1.1, 1.7, 2.5 and 3.0 folds higher than those in the Control group at 5, 10, 15 and 20 mJ/cm^2^, respectively (*P* < 0.01, Fig. 1B). Moreover, the mRNA and protein expression of PRDX1 were remarkably down-regulated in UVB-induced ARPE-19 cells. Notably, the low expression of PRDX1 was involved in high UVB intensity (*P* < 0.01, Fig. 1C/D). The above results suggested that UVB could inhibit ARPE-19 cell viability and induce cell damage, which might attributed to the down-regulation of PRDX1 expression.

### PRDX1 overexpression attenuates UVB-induced ARPE-19 cell damage

**Fig. 2:**
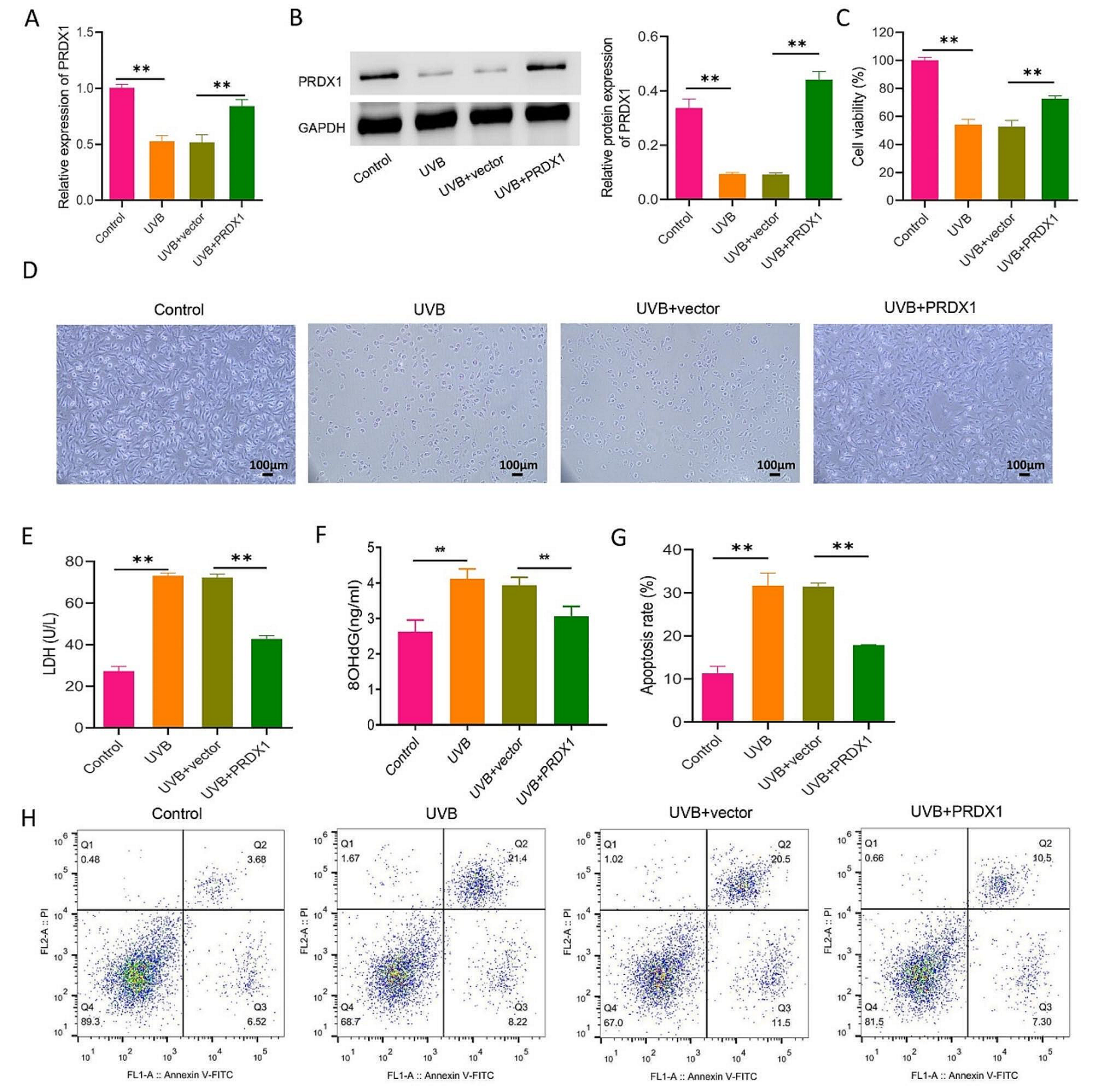
PRDX1 overexpression attenuates UVB-induced damage in ARPE-19 cells. **A**–**E**, qRT-PCR, western blotting, MTT and biochemical kits to determine the relative mRNA levels of PRDX1 (**A**), relative protein expression levels of PRDX1 (B), cell viability (**C**), cell morphology (**D**), and the levels of LDH (**E**) and 8-OHdG (**F**) in cell culture supernatant in ARPE-19 cells in the Control group, the UVB group, the UVB + vector group and the UVB + PRDX1 group, respectively. (**G/H**) Flow cytometry to evaluate the effect of UVB irradiation on apoptosis of ARPE19 cells that were untransfected or transfected with vector or PRDX1. **P < 0.01. PRDX1, peroxiredoxin 1; UVB: ultraviolet B; MTT, 3-[4,5-dimethylthiazol-2-yl]-2,5 diphenyl tetrazolium bromide; qRT-PCR, real-time quantitative reverse transcription polymerase chain reaction; LDH, lactate dehydrogenase; 8-OHdG: 8-hydroxy-2-deoxyguanosine

We increased the level of PRDX1 in UVB (20 mJ/cm^2^)-induced ARPE-19 cells by transfecting a PRDX1 overexpression vector to further demonstrate the relationship between UVB-induced ARPE-19 cell injury and down-regulation of PRDX1 expression. The results exhibited that as opposed to the Control group, the UVB group showed a significant downward trend in the expression level of PRDX1, with a 46% decrease in cell viability. Besides, the level of LDH and 8-OHdG was elevated in the culture supernatant by 1.7 times and 0.6 times, respectively (*P* < 0.01). In contrast, compared with the UVB + vector group, a remarkable elevation was observed in the expression level of PRDX1 in the UVB + PRDX1 group, while cell viability was increased by 38% in the cell culture supernatant, and the levels of LDH and 8-OHdG were decreased by 41% and 21%, respectively (*P* < 0.01, Fig. 2A–F). Furthermore, UVB induced cell apoptosis in ARPE-19 cells, and PRDX1 overexpression significantly inhibited UVB-induced cell apoptosis in ARPE19 cells (*P* < 0.05, Fig. 2G/H). The above results indicated that overexpression of PRDX1 attenuated UVB-induced ARPE-19 cell injury.

### PRDX1 overexpression inhibits UVB-induced oxidative stress in ARPE-19 cells

**Fig. 3:**
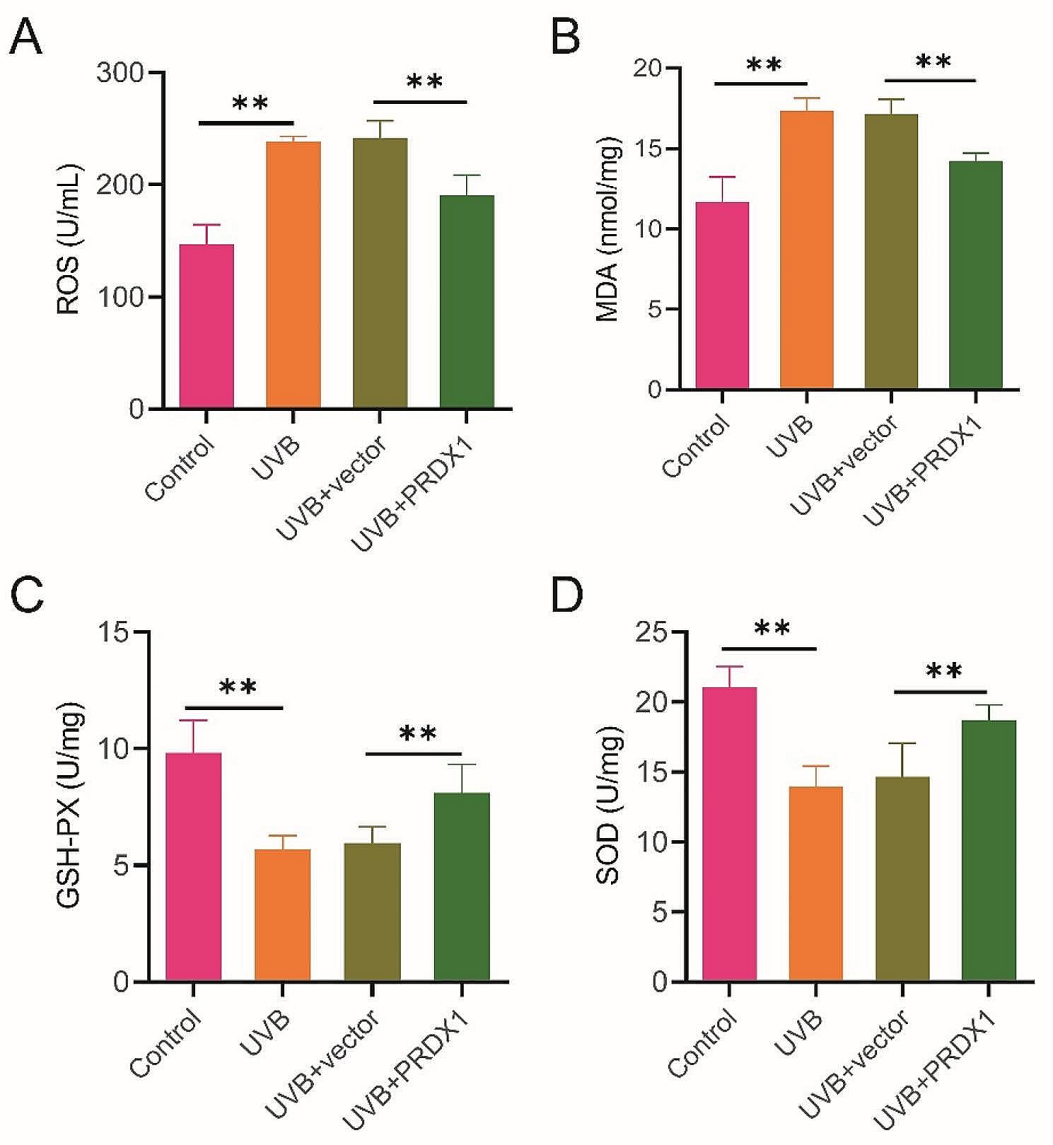
PRDX1 overexpression inhibits UVB-induced oxidative stress in ARPE-19 cells. A–D, the corresponding biochemical kits to detect the levels of ROS (**A**) and MDA (**B**), GSH-Px activity (**C**) and SOD activity (**D**) in the Control group, the UVB group, the UVB + vector group and the UVB + PRDX1 group, respectively, **P < 0.01. PRDX1, peroxiredoxin 1; UVB: ultraviolet B; ROS: reactive oxygen species; MDA, malondialdehyde; GSH-Px, glutathione peroxidase; SOD, superoxide dismutase

Subsequently, we examined the levels of oxidative stress-related factors in ARPE-19 cells in each group to investigate the effect of PRDX1 on UVB-induced oxidative stress response in ARPE-19 cells. The results revealed that the levels of ROS and MDA were considerably increased by UVB irradiation, and the activities of GSH-Px and SOD were markedly decreased in ARPE-19 cells in comparison with the Control group (*P* < 0.05). Therefore, UVB irradiation could induce oxidative stress in ARPE-19 cells. However, the levels of ROS and MDA were effectively dropped by overexpression of PRDX1, and the activities of GSH-Px and SOD were considerably raised in UVB-induced ARPE-19 cells (*P* < 0.05, Fig. 3A–D). Overall, UVB irradiation could induce oxidative stress in ARPE-19 cells, while overexpression of PRDX1 could alleviate UVB-induced oxidative stress.

### PRDX1 overexpression inhibits UVB-induced MAPK signaling pathway activity

**Fig. 4:**
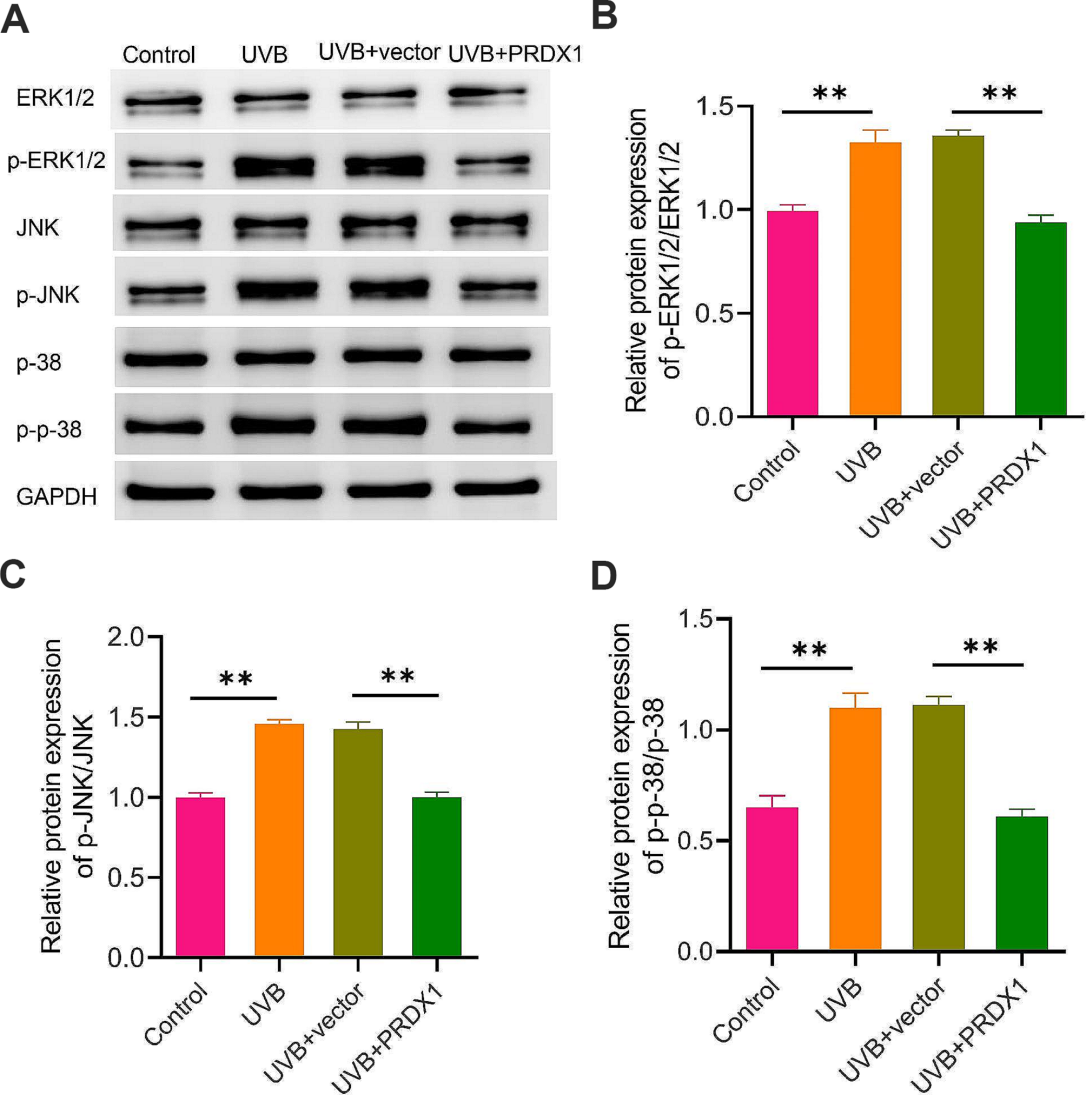
PRDX1 overexpression inhibits UVB-induced MAPK signaling activity. **A**–**D**, western blotting to measure the protein expression of ERK1/2, p-ERK1/2, JNK, p-JNK, p-38, and p-p38 in ARPE-19 cells (**A**) in the Control group, the UVB group, the UVB + vector group and the UVB + PRDX1 group, as well as the ratios of p-ERK1/2/ERK1/2 (**B**), p-JNK/JNK (**C**) and p-p38/p38 (**D**) in each group of cells, **P < 0.01. PRDX1, peroxiredoxin 1; UVB: ultraviolet B; MAPK: mitogen-activated protein kinase; ERK: extracellular signal-regulated kinase; p-ERK1/2, phosphorylated-ERK1/2; JNK, c-Jun N-terminal kinase

Ultimately, we detected the protein levels of mitogen-activated protein kinase (MAPK) signaling pathway-related proteins (ERK1/2, p-ERK1/2, JNK, p-JNK, p-38, and p-p38) in each group of cells by western blotting to further investigate the molecular mechanism of PRDX1 in UVB-induced injury of ARPE-19 cells. The results displayed that the levels of p-ERK1/2, p-JNK, and p-p38 were higher in the UVB group, and the ratios of p-ERK1/2/ERK1/2, p-JNK/JNK, and p-p38/p38 were larger relative to the Control group (*P* < 0.05). The above results indicated that UVB irradiation could significantly activate the MAPK signaling pathway in ARPE-19 cells. However, overexpression of PRDX1 could notably reduce the levels of p-ERK1/2, p-JNK and p-p38 in UVB-irradiated ARPE-19 cells and the ratios of p-ERK1/2/ERK1/2, p-JNK/JNK and p-p38/p38 (*P* < 0.05, Fig. 4A–D). To sum up, UVB irradiation could induce the activation of the MAPK signaling pathway in ARPE-19 cells and PRDX1 might play a protective role in UVB-induced ARPE-19 cell injury by inhibiting the activity of this pathway.

## Discussion

AMD is a leading cause of severe vision loss and blindness among the elderly population in developed countries [19]. AMD is characterized by dysfunction and eventual loss of RPE cells, which is related to oxidative and nitrosative damage [16]. In addition, photodamage can also lead to retinal cell damage and even apoptosis, which is one of the key causes contributing to the development of AMD [20]. Overexposure to UV radiation is associated with the production of ROS. ROS can promote oxidative damage to RPE and the onset of AMD [21]. LDH is one of the markers of cytotoxicity, and its activity is highly negatively correlated with cell survival [22]. In this study, the viability of ARPE-19 cells was reduced by UVB irradiation at various intensities, while the level of LDH leakage was increased in the cell culture medium. Cell viability decreased with increasing UVB intensity. while LDH level was positively correlated with UVB intensity. These results suggested that UVB irradiation exerted cytotoxic effects on ARPE-19 cells. Besides, Oh et al., uncovered that cell activity of ARPE-19 cells was significantly decreased by UVB radiation at different intensities [23], which is consistent with our results.

PRDX1 is a peroxidase molecule that is abundant in prokaryotes and eukaryotes. It plays a protective role in various diseases such as aging, acute tissue injury, neurodegenerative diseases and cancer, by regulating multiple ROS-related signaling pathways [24]. Overexpression of PRDX1 has been reported to decrease the levels of ROS, thereby alleviating oxidative stress [24, 25]. In this study, UVB irradiation significantly inhibited PRDX1 expression in RPE cells, with its expression being negatively correlated with UVB intensity. After transfection of PRDX1, the viability of UVB-induced ARPE-19 cells was restored and the LDH level was considerably dropped. Therefore, overexpression of PRDX1 could alleviate UVB-induced photodamage. Similarly, Cai et al., discovered that the expression of PRDX1 was significantly down-regulated in UVA-irradiated RPE cells [16]. However, there are no studies on the role of PRDX1 in UVB-induced RPE cells.

The pathogenesis of UVB-induced photodamage has been reported to be closely associated with oxidative stress. The main relevant indicators of oxidative stress response include ROS, SOD, GSH-Px and MDA. Among them, endogenous SOD produced by the body, can scavenge free radicals and eliminate excessive ROS. The level of SOD can reflect the body’s capacity to resist oxidative stress. When ROS levels rise, antioxidant molecules react to neutralize them. However, an excessive increase can lead to redox imbalance, which will contribute to the production of cytotoxicity, envelope lipid peroxidation, creation of MDA, and aggravation of oxidative stress [26]. The up-regulation of PRDX1 in cells and tissues under oxidative stress is considered one of the cellular recovery reactions after oxidative damage [27]. Jiang et al. pointed out that PRDX1 overexpression inhibits doxorubicin-induced oxidative stress by increasing SOD and CAT activities, decreasing MDA content and nicotinamide adenine dinucleotide phosphate oxidase activity, thus alleviating cardiac toxicity [24]. As demonstrated by Kang et al., the levels of GSH and SOD were decreased in PRDX1 gene-silenced cells [28]. In this study, the results indicated that UVB induced oxidative stress in ARPE-19 cells, which aligns with the findings of an earlier study [23]. PRDX1 overexpression significantly reduced the levels of ROS and MDA and elevated the activity of GSH-Px and SOD, thus alleviating UVB-induced oxidative stress. Therefore, we speculated that PRDX1 might act as a protective role by preventing UVB-induced oxidative stress in RPE cells.

Oxidative stress is regulated by several signaling pathways. MAPK signaling activation has been involved in ROS-induced apoptosis in RPE cells [29]. The MAPK signaling process, a typical three-component enzymatic cascade reaction, participates in the regulation of cell development. ERK, p38 and JNK constitute the core components of MAPKs [30]. Among them, JNK and p38 are multiple extracellular signals with similar functions. Their substrate phosphorylation is associated with cell inflammation, apoptosis, growth cycle and differentiation. In particular, they are involved in regulating the release of inflammatory mediators, while ERK primarily participates in cell growth and differentiation [31]. UVB exposure has been uncovered to enhance the MAPK signaling pathway and phosphorylation of the MAPK pathway, including ERK, JNK, and p38 phosphorylation [29]. In this study, UVB was also found to induce the phosphorylation of the MAPK pathway in cells. Additionally, the levels of ERK, JNK and p38 phosphorylation were inhibited in cells after PRDX1 overexpression. Therefore, PRDX1 overexpression inhibited UVB-induced MAPK signaling activation.

Overall, PRDX1 plays a protective role in UVB-induced ARPE-19 cell damage, potentially by inhibiting oxidative stress and suppressing the activation of the MAPK signaling pathway. The results provide a theoretical basis for PRDX1 as a potential target for AMD treatment. However, there are some limitations in this study. For instance, the MAPK pathway was not validated through the corresponding pathway activators or inhibitors, so we could not fully prove that PRDX1 plays a role in inhibiting oxidative stress and alleviating cell damage through the MAPK pathway. Moreover, no relevant in vivo experiments were carried out, and all of the conclusions given in this study were from in vitro experiments. Therefore, relevant in vivo experiments are needed to verify the findings of this study in the future.

### Electronic supplementary material

Below is the link to the electronic supplementary material.

Supplementary Material 1

## Data Availability

The datasets used and/or analyzed during the current study are available from the corresponding author on reasonable request.
