# Supplementary material for: PRDX1 exerts a photoprotection effect by inhibiting oxidative stress and regulating MAPK signaling on retinal pigment epithelium
Source: BMC Ophthalmol. 2024 Jun 6;24:237. doi: 10.1186/s12886-024-03489-4 (PMC11155104; doi:10.1186/s12886-024-03489-4)

Figure 1D

The original Western Blot images of PRDX1 in Figure 1D. From left to right: Control, 5, 10, 15, 20

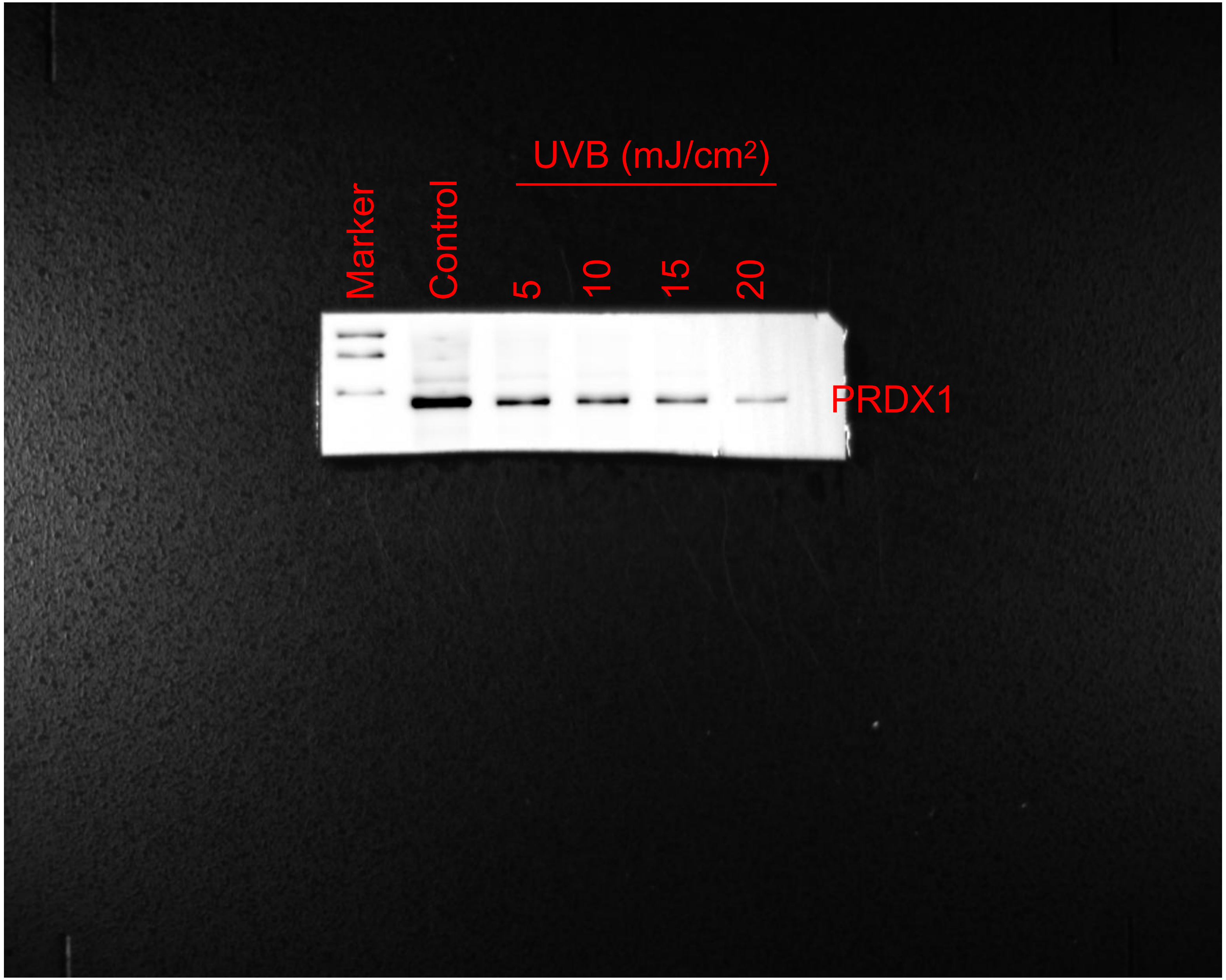

Figure 1D

The original Western Blot images of GAPDH in Figure 1D. From left to right: Control, 5, 10, 15, 20

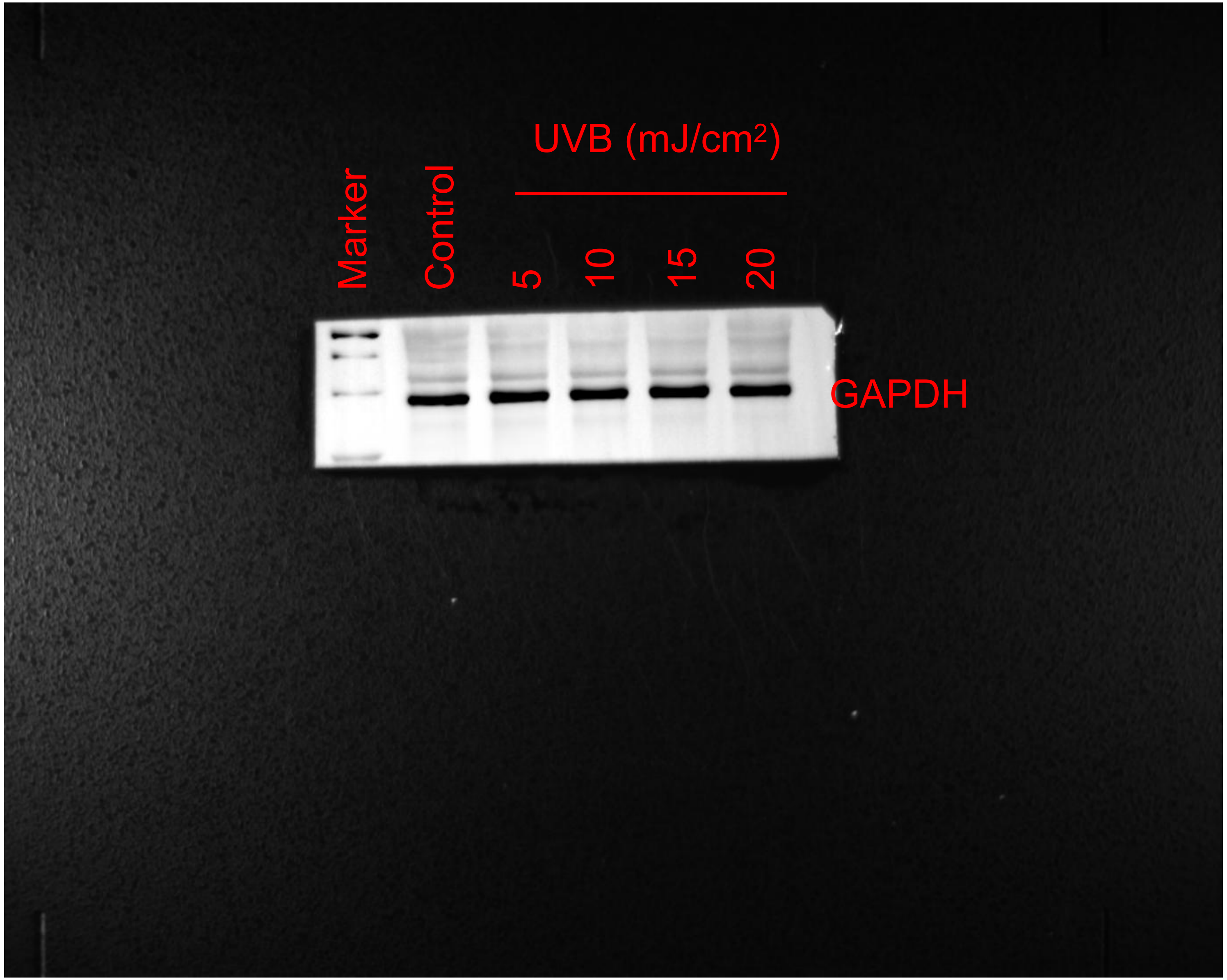

Figure 2B

The original Western Blot images of PRDX1 in Figure 2B. From left to right: Control, UVB, UVB+vector, UVB+PRDX1

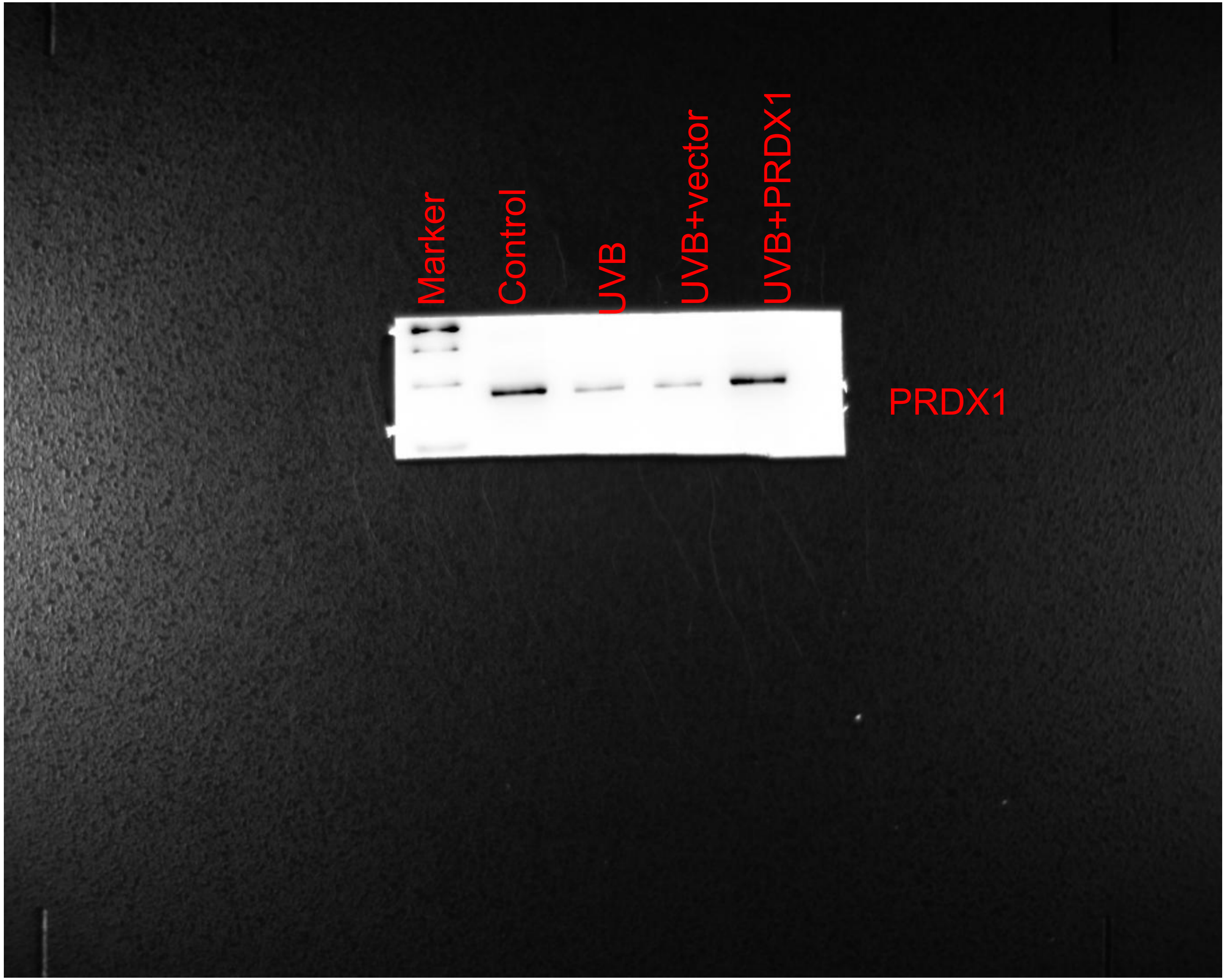

Figure 2B

The original Western Blot images of GAPDH in Figure 2B. From left to right: Control, UVB, UVB+vector, UVB+PRDX1

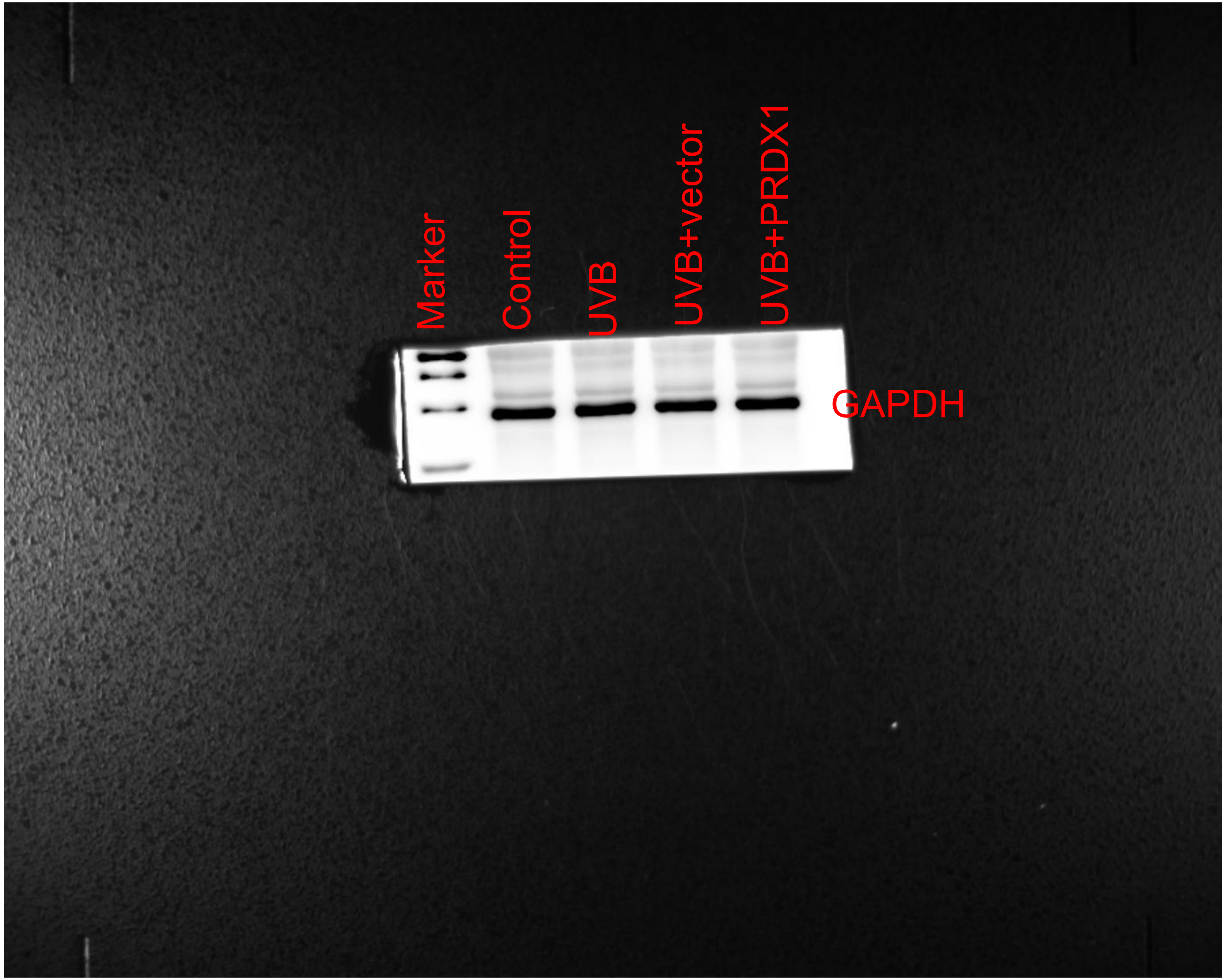

Figure 4

The original Western Blot images of ERK 1/2 in Figure 4. From left to right: Control, UVB, UVB+vector, UVB+PRDX1

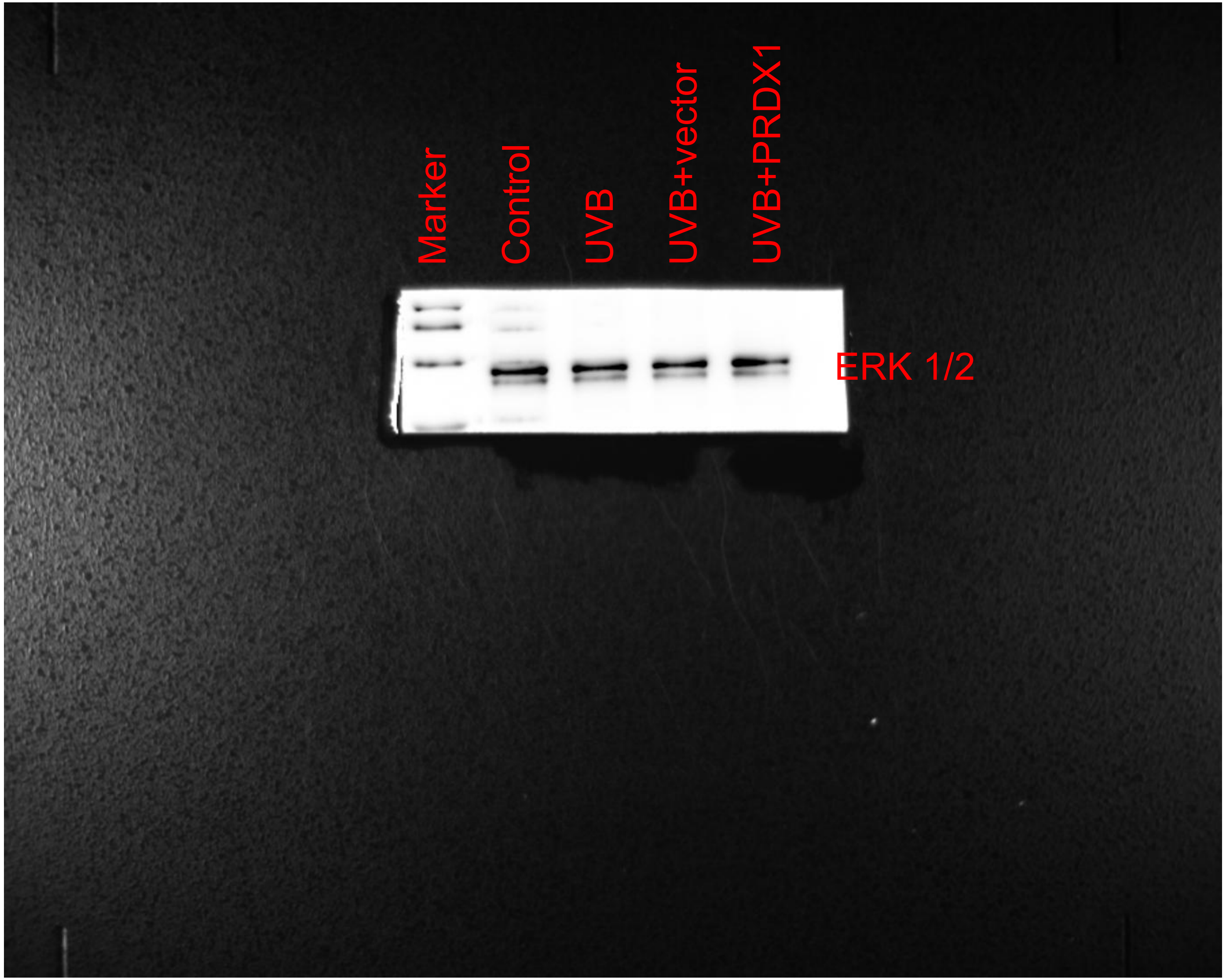

Figure 4

The original Western Blot images of p-ERK 1/2 in Figure 4. From left to right: Control, UVB, UVB+vector, UVB+PRDX1

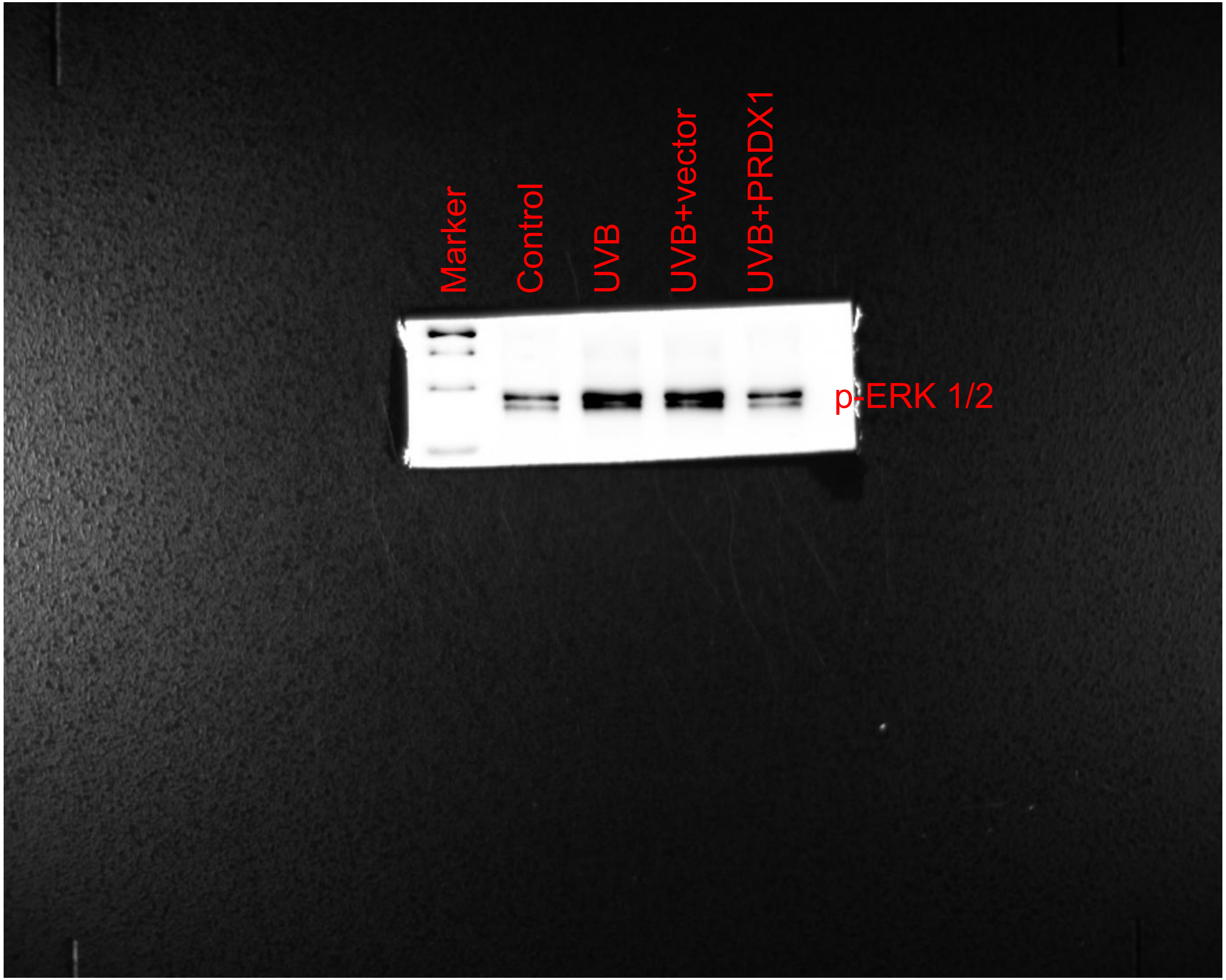

Figure 4

The original Western Blot images of JNK in Figure 4. From left to right: Control, UVB, UVB+vector, UVB+PRDX1

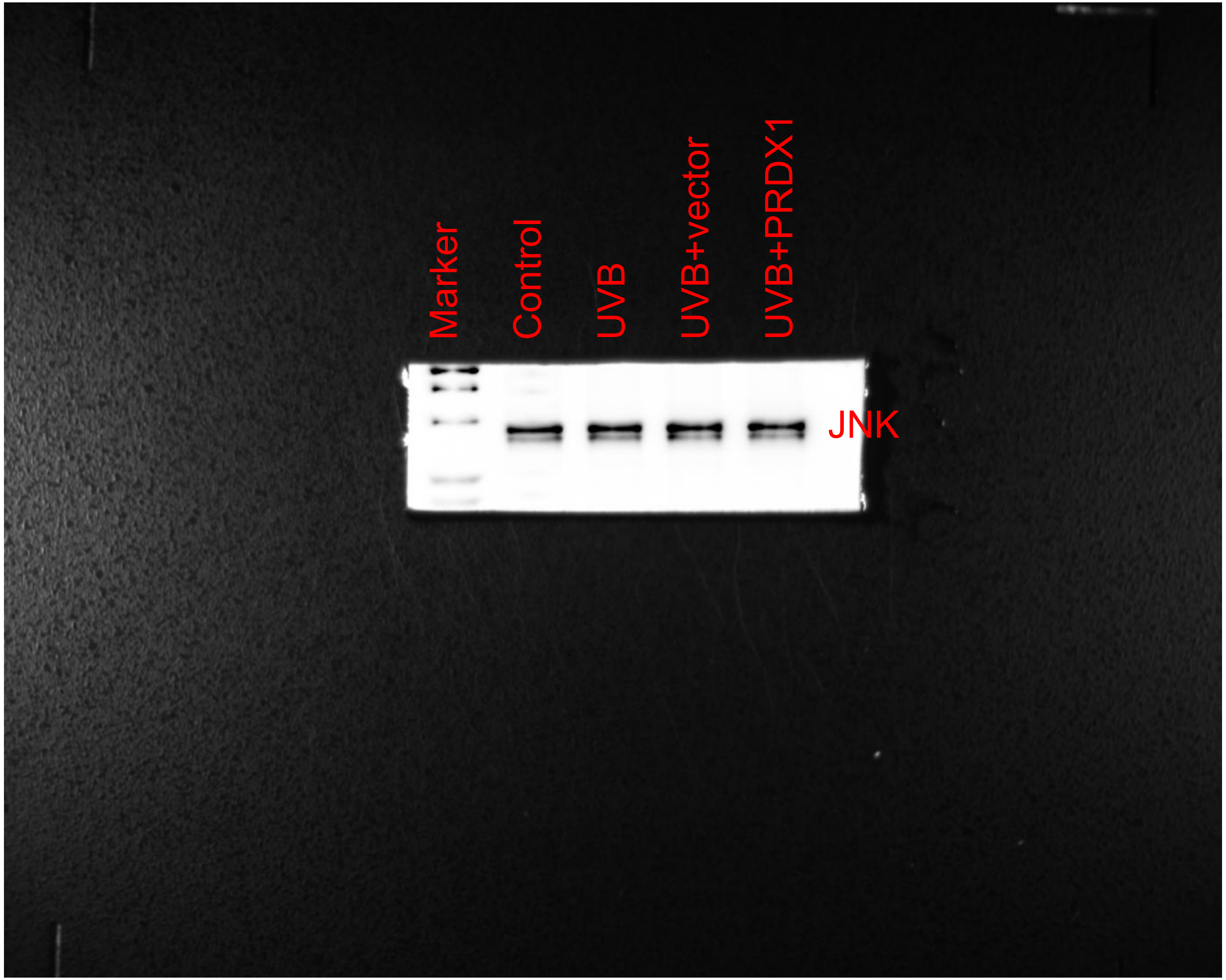

Figure 4

The original Western Blot images of p-JNK in Figure 4. From left to right: Control, UVB, UVB+vector, UVB+PRDX1

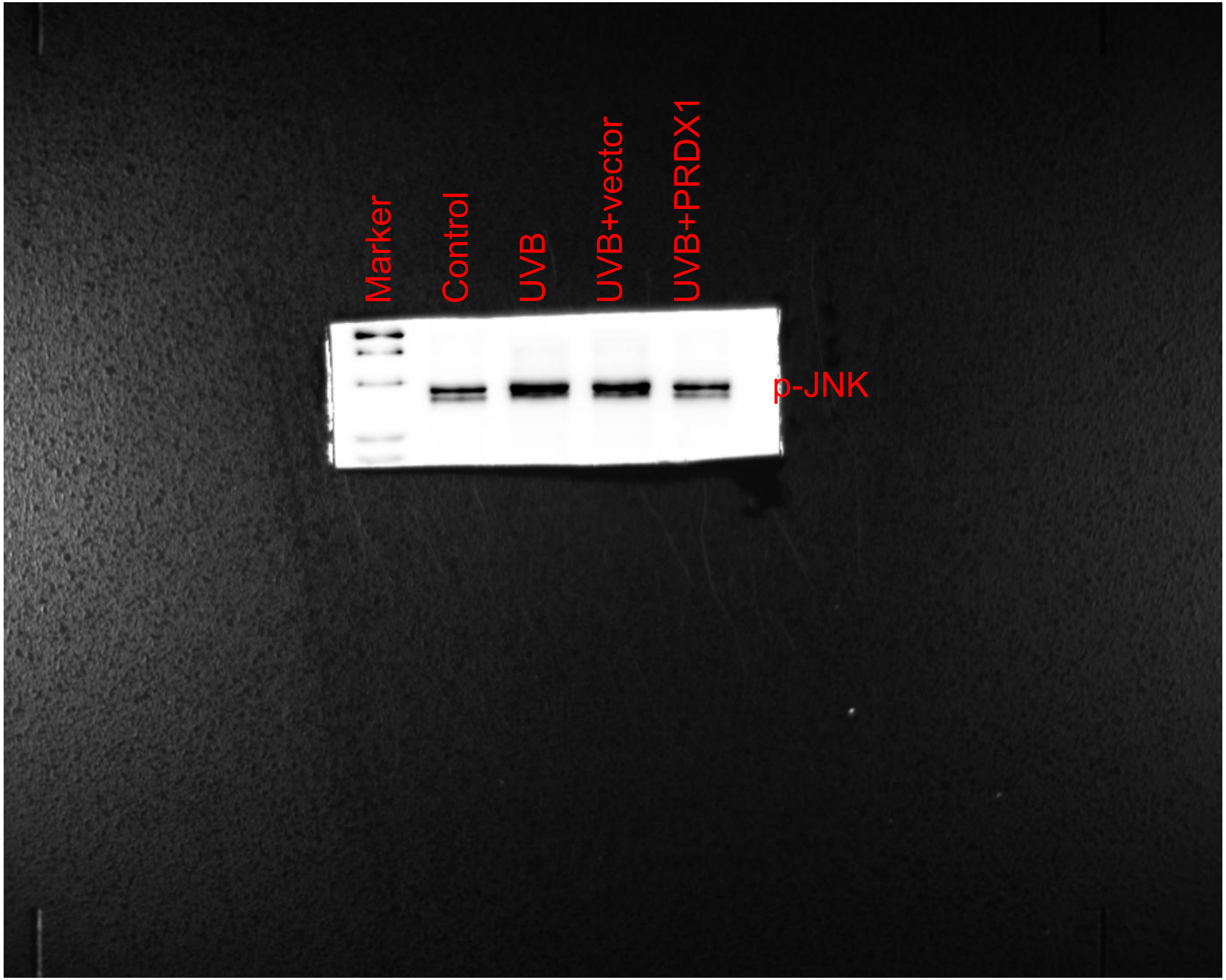

Figure 4

The original Western Blot images of p-38 in Figure 4. From left to right: Control, UVB, UVB+vector, UVB+PRDX1

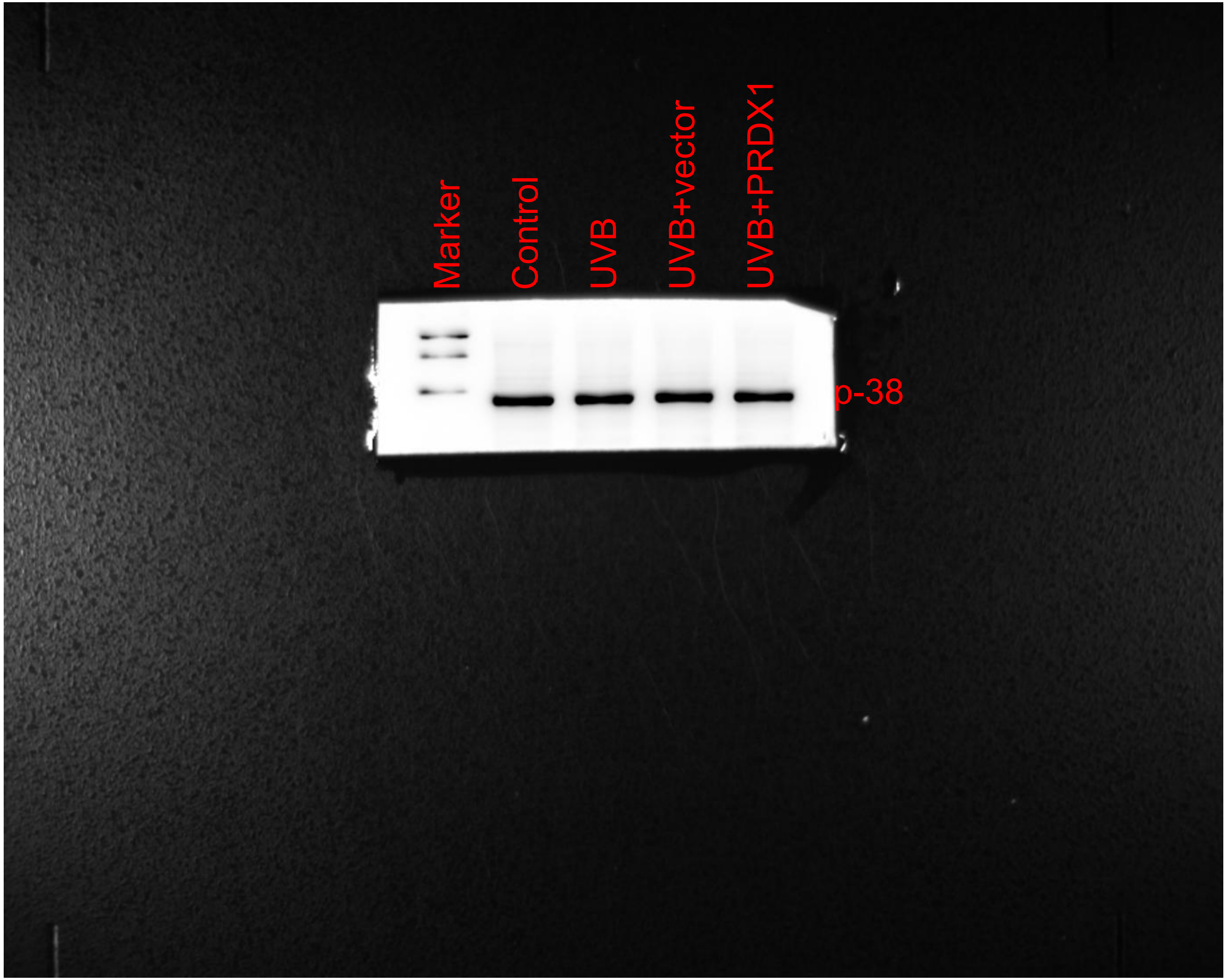

Figure 4

The original Western Blot images of p-p-38 in Figure 4. From left to right: Control, UVB, UVB+vector, UVB+PRDX1

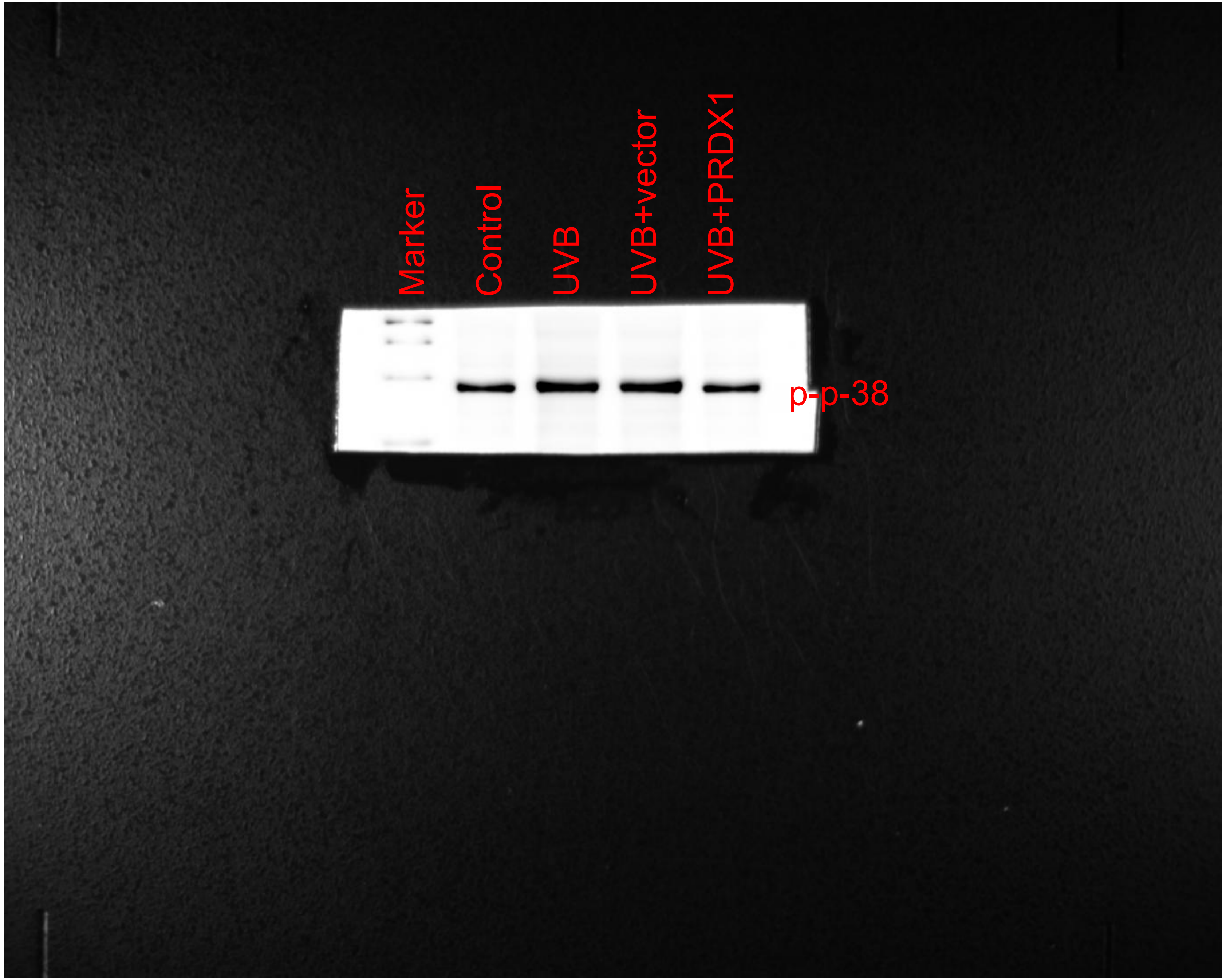

Figure 4

The original Western Blot images of GAPDH in Figure 4. From left to right: Control, UVB, UVB+vector, UVB+PRDX1

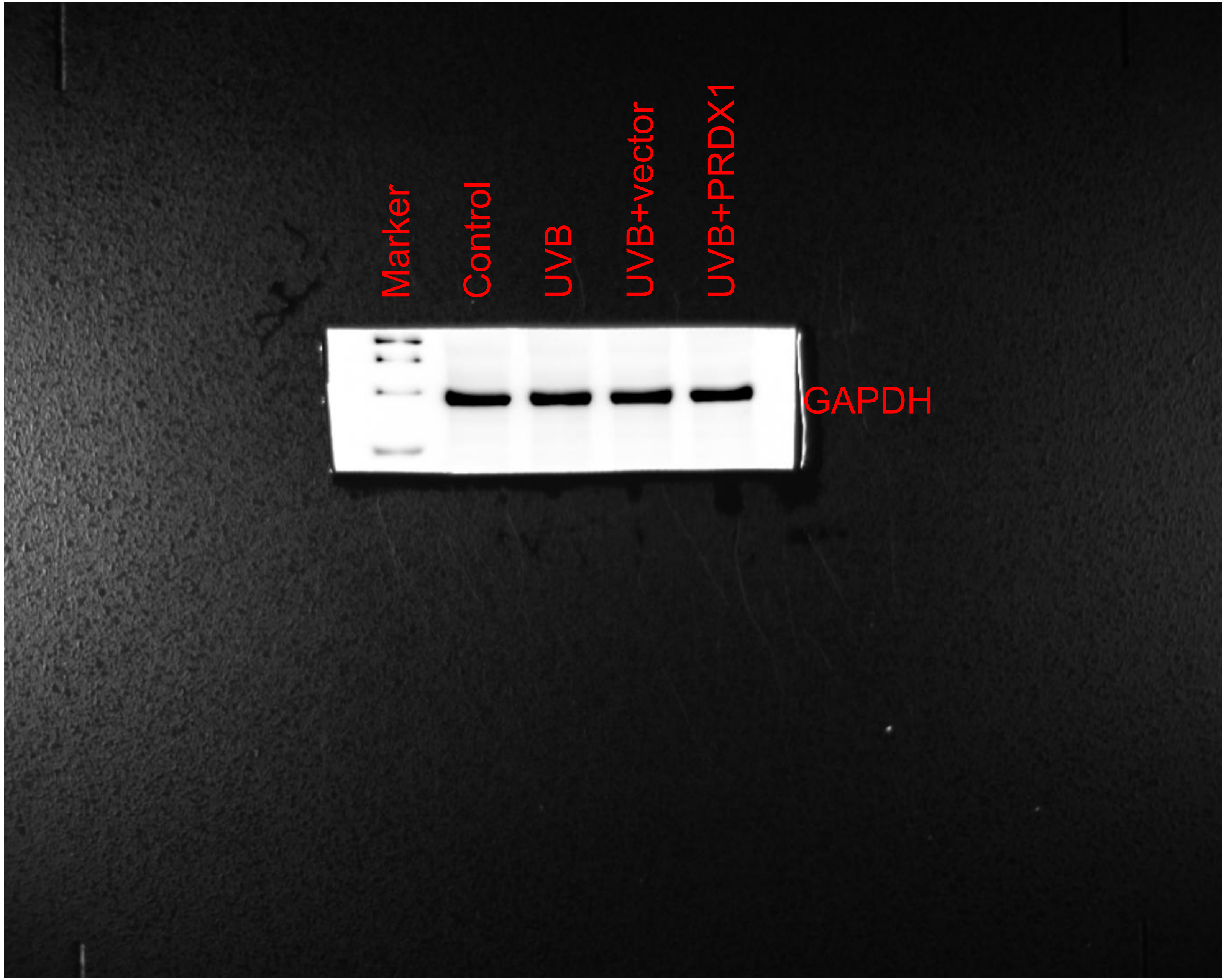

Supplement: Supplementary file 1 — Supplementary Material 1 [file 12886_2024_3489_MOESM1_ESM.pdf]
